# Feasibility of Asynchronous and Automated Telemedicine in Otolaryngology: Prospective Cross-Sectional Study

**DOI:** 10.2196/23680

**Published:** 2020-10-19

**Authors:** Dongchul Cha, Seung Ho Shin, Jungghi Kim, Tae Seong Eo, Gina Na, Seonghoon Bae, Jinsei Jung, Sung Huhn Kim, In Seok Moon, Jaeyoung Choi, Yu Rang Park

**Affiliations:** 1 Department of Otorhinolaryngology Yonsei University College of Medicine Seoul Republic of Korea; 2 Department of Biomedical Systems Informatics Yonsei University College of Medicine Seoul Republic of Korea

**Keywords:** telemedicine, otolaryngology, otology, automated diagnosis, asynchronous, COVID-19, diagnosis, feasibility, cross-sectional

## Abstract

**Background:**

COVID-19 often causes respiratory symptoms, making otolaryngology offices one of the most susceptible places for community transmission of the virus. Thus, telemedicine may benefit both patients and physicians.

**Objective:**

This study aims to explore the feasibility of telemedicine for the diagnosis of all otologic disease types.

**Methods:**

A total of 177 patients were prospectively enrolled, and the patient’s clinical manifestations with otoendoscopic images were written in the electrical medical records. Asynchronous diagnoses were made for each patient to assess Top-1 and Top-2 accuracy, and we selected 20 cases to conduct a survey among four different otolaryngologists to assess the accuracy, interrater agreement, and diagnostic speed. We also constructed an experimental automated diagnosis system and assessed Top-1 accuracy and diagnostic speed.

**Results:**

Asynchronous diagnosis showed Top-1 and Top-2 accuracies of 77.40% and 86.44%, respectively. In the selected 20 cases, the Top-2 accuracy of the four otolaryngologists was on average 91.25% (SD 7.50%), with an almost perfect agreement between them (Cohen kappa=0.91). The automated diagnostic model system showed 69.50% Top-1 accuracy. Otolaryngologists could diagnose an average of 1.55 (SD 0.48) patients per minute, while the machine learning model was capable of diagnosing on average 667.90 (SD 8.3) patients per minute.

**Conclusions:**

Asynchronous telemedicine in otology is feasible owing to the reasonable Top-2 accuracy when assessed by experienced otolaryngologists. Moreover, enhanced diagnostic speed while sustaining the accuracy shows the possibility of optimizing medical resources to provide expertise in areas short of physicians.

## Introduction

COVID-19, which was declared a pandemic by the World Health Organization, has shifted societies toward noncontact. Since the disease is highly transmissible between humans and often has respiratory symptoms [1], otolaryngologists are among the most susceptible physicians for infection. Hospital visits raise the risk of hospital-acquired COVID-19 infections, which calls for telemedicine. Currently, telemedicine is widely deployed in the United States and is on the rise [2]. Telemedicine can be synchronous or asynchronous [3]. For example, in otolaryngology, a Veterans Affairs model uses a community-based outpatient clinic to connect with an otolaryngologist [4]. It is similar to walk-in office clinics in that a clinic visit happens in real time. However, from the physician’s point of view, connecting and interviewing the patient in real time through videoconferencing is likely to cause a temporal overhang compared to meeting the patient in person and, therefore, is less efficient. In an asynchronous model, the patient’s information and physical findings are presented to the physician as medical records, and therefore, further interview with the patient is not possible. It is more prevalent in consultations between health care providers, and a study by Liddy et al [5] showed it to improve access to specialists. In otology, a study with asynchronous video-otoscopy taken by a tele-health facilitator reported a diagnostic capability equivalent to direct otoscopy by physicians [6].

Diagnosing otologic diseases is mostly noninvasive; initial diagnosis usually involves detailed history taking and physical examination by otoscopy. The problem of telemedicine in the field of otology lies in the presentation of physical inspection. Videoconferencing methods enable physicians to see lesions on the skin; however, looking inside the external ear canal and at the eardrums requires a dedicated imaging device. Conventional otoscopy is not expensive, but sharing raw images is not possible. To share an otoscopic image, one has to rely on expensive otoendoscopic imaging systems. In a recent study, smartphone-enabled otoscopy was shown to be just as effective as conventional otoscopy [7], showing promising possibilities of telemedicine. Nowadays, some consumer-grade tools are more affordable, costing under $40 [8]. With more accessibility to consumer-grade otoendoscopes, the possibility of patients directly contacting physicians for medical advice is increasing, especially during the COVID-19 pandemic era. We, therefore, evaluated the possibility of using telemedicine in otology.

Previous studies focused mainly on findings of otoendoscopy [6,9,10], which did not include other ear diseases such as dizziness, facial palsy, and tumors. In this study, we target all otologic diseases to explore the possibility of converting the operations of the entire otology clinical office from offline to online. Accordingly, we prospectively performed a telemedicine scenario for every new visitor to the otology department clinic. First, we compared the accuracy and likelihood of remote diagnosis to that of office walk-in visits. Second, we conducted a diagnostic survey among otolaryngologists to explore interrater reliability and measure the speed of diagnosis as measures of diagnostic consistency and efficiency. Finally, we created a decision tree to perform an automated diagnostic system, using our previously created otoendoscopic image classification system [11], and compared its accuracy and speed to those of otolaryngologists.

## Methods

### Patient Selection

**Figure 1:**
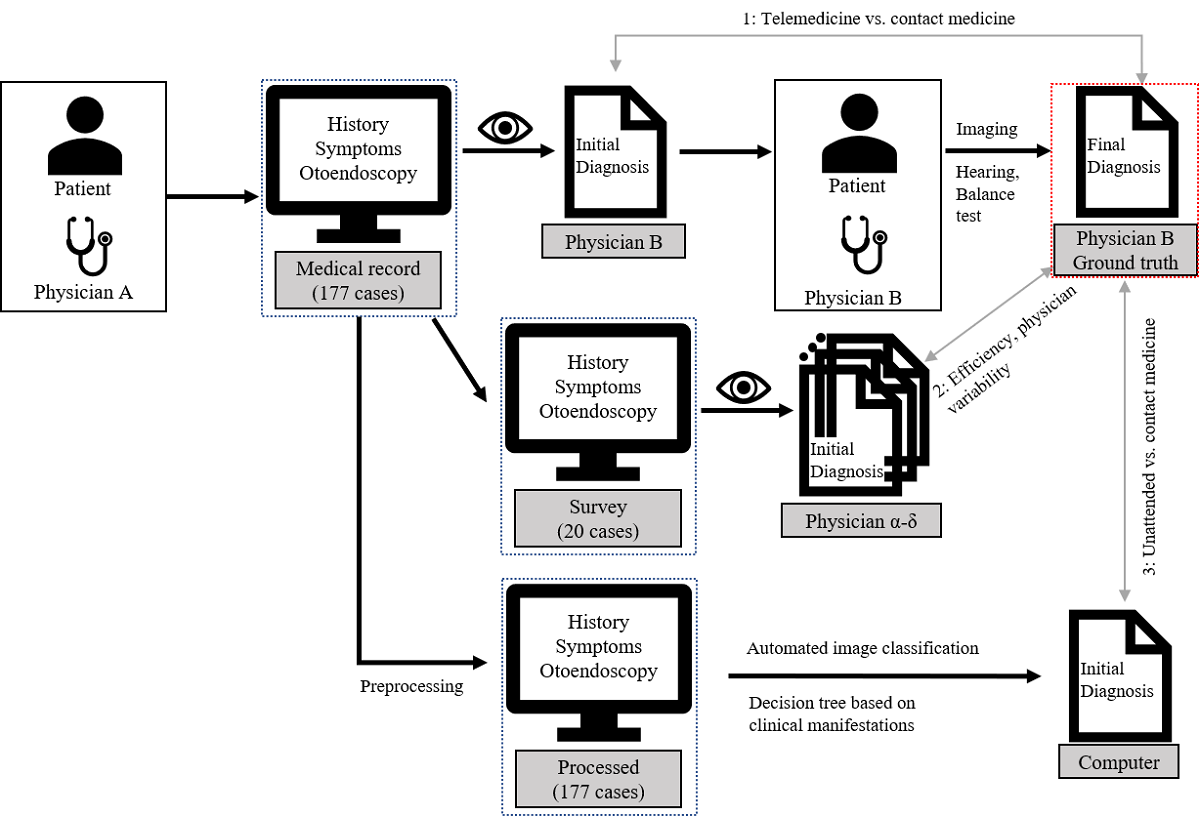
Summary of three telemedicine scenarios in this study. Otoendoscopic images were acquired with a consumer-grade device. The eye sign indicates that the records were viewed by corresponding physicians. Physician A is the otolaryngologist or otolaryngology resident, Physician B is the attending physician (otolaryngologist), and Physician α-δ is the otolaryngologist.

All first-time visitors to the Severance Hospital’s Otolaryngology Outpatient Clinic from June 8, 2020, to June 19, 2020, who were 18 years or older and having otologic symptoms, were prospectively recruited for the study. A total of 201 patients were eligible for the study. However, 17 patients refused to enroll in the study, and we excluded 2 patients who had Alzheimer disease and one with a known genetic disease (Usher syndrome). There were 4 patients who were workers inside the hospital. They were excluded because of their vulnerability to the rejection of the study. Therefore, 177 patients were included in the final analysis. The Severance Hospital’s review board approved this study (Institutional Review Board no 4-2020-0428), and written consent was obtained from all participants. All methods were performed following the Declaration of Helsinki (1964). Figure 1 shows the study design.

### Asynchronous Telemedicine Versus Contact Medicine

After obtaining written consent, an otolaryngologist or otolaryngology resident recorded the detailed patient history, noted the clinical manifestations, and acquired an otoendoscopic image of the eardrum and the external auditory canal using a consumer-grade otoendoscopy device tethered to an Android smartphone. In cases of facial palsy or external ear diseases, the default camera app on the Android smartphone was used to acquire facial or lesional photos. The written medical record mainly included chief complaints, duration, present illness, and medico-surgical history. Additional structured profiles, including onset, duration, characteristics, and aggravating and relieving factors, were filled out if the patient had dizziness. All this information and the otoendoscopic image were placed into the electronic medical record. The attending physician then made an initial diagnosis with the information in the electronic medical record before interviewing the patient, thus mimicking an asynchronous telemedicine setting. After the initial impression was set, the patient walked into the office for the attending physician to perform a more detailed history taking; take images using a professional-grade otoendoscope; and perform a physical examination, audiologic tests, and imaging studies if necessary. Based on these, the attending physician made a final diagnosis. Attending physicians were allowed to make no more than initial and final impressions. Of note, our clinic is a tertiary referral center. Still, referral letters and additional study results from previous clinics were not seen by the attending physician when making the initial impression. We should also mention that the patients’ reservations were assigned mainly according to the attending physician’s subspecialty (acoustic tumors, dizziness, or hearing rehabilitation); therefore, to some extent, the attending physician had an advantage of anticipating the patient’s diagnosis.

### Efficiency and Interphysician Variability in Telemedicine Diagnosis

An online survey was conducted with four otolaryngologists to evaluate the diagnostic variability between physicians. The survey was conducted in an open type question to reflect real-life clinical settings. Each surveyee was allowed to make up to two diagnoses based on the written patient information, otoendoscopic images acquired with the consumer-grade otoendoscopy device, and photos of facial expressions or lesions acquired with the built-in camera app of the smartphone, if applicable. There were 20 patients that were randomly selected from the 177 patients, and all surveyees evaluated them in the same order. When answering the questionnaire, we timed the total test time to assess the speed of diagnosis. Diagnostic accuracy was calculated as Top-1 and Top-2 accuracy using the Cohen kappa method. Diagnostic speed was measured as the number of diagnoses per minute.

### Unattended (Automated) Versus Contact Medicine and Asynchronous Telemedicine Diagnoses

An automated diagnosis decision tree–based model was fed with the patient’s symptoms and otoendoscopic images. Otoendoscopic images were classified into normal, tympanic membrane perforation, attic retraction, myringitis, otitis media with effusion, and tumors using an automated otoendoscopic image classification model [11]. Since the automated classification model could not handle facial palsy and preauricular sinuses, these were manually marked as a correct diagnosis, as all surveyees had correctly identified these diseases. The decision tree was based on single-label classification, so only Top-1 was used for comparison of accuracy and likelihood of diagnosis (Cohen kappa). The speed of diagnosis was measured as the total execution time when running under a system environment of Intel Core i7-8750H (Intel Corporation), 16 GB of RAM, and GeForce RTX 2070 (Nvidia Corporation). We converted runtime to a scale of diagnoses per minute.

### Classification of the Diseases and Evaluation Metrics

The agreement between telemedicine (initial impression) and contact medicine (final impression) was assessed by Top-1 and Top-2 accuracies. The diagnosis was categorized into 21 categories, based on the International Classification of Diseases, 10th revision diagnostic hierarchy [12]. Accordingly, the next necessary clinical steps were indicated. For example, in cases of hearing impairment, we additionally categorized sudden onset, since the treatment strategy (steroids administration) differs from typical deafness (hearing rehabilitation). Likewise, we divided patients with suspected peripheral vestibulopathy as having benign paroxysmal positional vertigo, vestibulopathy, or Meniere disease. The agreement between the initial and final diagnoses was measured using the Cohen kappa method [13]. The kappa (κ) scores were interpreted as follows: κ<0, poor; 0.01-0.20, slight; 0.21-0.40, fair; 0.41-0.60, moderate; 0.61-0.80, substantial; and 0.81-1, near perfect agreement [14].

### Statistical Analysis

The Cohen kappa method was used to calculate the diagnostic accuracy and the likelihood of agreement in the diagnosis between survey participants, using the Scikit-learn python package [15]. Continuous variables are presented as mean and standard deviation.

## Results

### Patient Distribution

There were 177 patients in total, ranging in age from 19 to 95 years (69 male and 108 female; mean 55.57, SD 17.05 years). The distribution of diseases in these patients is summarized in Table 1. Hearing impairment was the most common cause of visits, followed by dizziness and chronic otitis media.

**Table 1:** Diagnoses during contact medicine.

Diagnosis	Count (n=177), n
Hearing loss	41
Chronic otitis media	25
Benign paroxysmal positional vertigo	23
Sudden sensorineural hearing loss	19
Vestibulopathy	16
Tinnitus	14
External ear disease (preauricular fistula, otohematoma)	7
Meniere disease	5
Normal finding	5
Bell palsy	4
Schwannoma (vestibular, facial)	4
Eustachian tube dysfunction	3
Cerumen impaction	3
Middle ear tumors	2
Otitis media with effusion	2
Acute otitis media	1
Postoperative complication (cochlear implant device exposure)	1
Traumatic eardrum perforation	1
Superior canal dehiscence syndrome or perilymph fistula	1

Contact medicine included additional diagnostic modalities (audiologic test, imaging, vestibular function tests) if necessary and was considered as the ground-truth label.

### Asynchronous Telemedicine Versus Contact Medicine

We evaluated the accuracy of telemedicine versus contact medicine. The mean Top-1 and Top-2 accuracies were 83.05% and 88.14%, respectively. Of the 177 patients, 23 had a second likely diagnosis, accounting for the difference between mean Top-1 and Top-2 accuracies. We individually calculated each diagnostic class sensitivity in the two most likely diagnoses in the telemedicine setting (Table 2). Diseases with strong clinical correlations, such as sudden sensorineural hearing loss, had 100% sensitivity. In addition, apparent findings such as external ear disease and facial palsy had 100% sensitivity. Diseases related to dizziness had relatively low sensitivity due to the absence of the required physical examination results, such as nystagmus tests, at the time of electronic medical record creation. Vestibular or facial nerve schwannoma is diagnosed by magnetic resonance imaging, not by physical examination; therefore, low sensitivity was inevitable. All predictions and ground-truth labels are presented as a confusion matrix in Multimedia Appendix 1.

**Table 2:** Telemedicine sensitivities in diagnosing different otological diseases (Top-2).a

Diagnoses	Sensitivity (%)
Hearing loss	100.00
Sudden sensorineural hearing loss	100.00
External ear disease	100.00
Facial palsy	100.00
Chronic otitis media	95.83
Tinnitus	80.00
Meniere disease	80.00
Benign paroxysmal positional vertigo	79.17
Schwannoma	75.00
Vestibular neuritis	73.33
Normal finding	60.00

^a^Incidences of less than 4 were excluded.

### Efficiency and Interphysician Variability in Telemedicine Diagnosis

Four otolaryngologists reviewed 20 randomly selected cases in the same order (Table 3). The average Top-1 and Top-2 accuracies were 76.25% (SD 10.31%) and 91.25% (SD 7.50%), respectively. The mean Top-2 accuracy in this assessment was higher than the asynchronous telemedicine’s mean Top-2 accuracy for the entire cohort (86.44% vs 91.25%). In the questionnaire, the surveyees were more likely to add a second probable diagnosis, compared to the original telemedicine scenario. Out of the 177 patients, there were 23 (13.0%) cases with a second-likely diagnosis assessed in the original telemedicine scenario. In the questionnaire assessment, there were 8 out of 20 (mean 40.0%, SD 21.21%) second-line impressions on average. Therefore, despite the Top-1 accuracy being lower, the Top-2 accuracy was higher in the survey scenario. Interrater variability was also assessed (Table 4). Substantial agreement was present among the four surveyees (κ=0.71) when only the first-line diagnosis was considered. An almost perfect agreement was observed (κ=0.91) when considering whether the survey participants agreed on one of the first-line or second-line diagnoses. Answering 20 questionnaires took an average of 14 minutes and 2 seconds (SD 20 seconds), making an average of 1.55 (SD 0.48) diagnoses per minute.

**Table 3:** Accuracies and diagnostic speed in different approaches.

Approach		Top-1 (%)	Top-2 (%)	Diagnoses/min
Asynchronous telemedicine		77.40	86.44	N/A^a^
**Survey of 20 cases, average**		76.25	91.25	1.55
	Otologist 1	75.00	85.00	1.63
	Otologist 2	90.00	100.00	1.51
	Otologist 3	65.00	85.00	2.11
	Otologist 4	75.00	95.00	0.95
Automated system		69.50	N/A	667.90

^a^N/A: not applicable.

**Table 4:** Interrater reliability among four otolaryngologists based on Cohen kappa in Top-1 and Top-2.

Diagnoses		Cohen kappa
**Top-1**		
	Overall	0.71
	External ear disease	1.00
	Postoperative complication	1.00
	Cerumen impaction	1.00
	Superior semicircular canal dehiscence syndrome	1.00
	Benign paroxysmal positional vertigo	0.82
	Tinnitus	0.82
	Otitis media with effusion	0.82
	Eustachian tube dysfunction	0.82
	Facial palsy	0.82
	Chronic otitis media	0.69
	Acute otitis media	0.37
	Hearing loss	0.19
	Meniere disease	0.18
	Normal findings	0.18
**Top-2**		
	Overall	0.91
	Middle ear tumor	1.00
	Benign paroxysmal positional vertigo	1.00
	Vestibular neuritis	1.00
	Tinnitus	1.00
	Meniere disease	1.00
	External ear disease	1.00
	Postoperative complication	1.00
	Cerumen impaction	1.00
	Superior semicircular canal dehiscence syndrome	1.00
	Facial palsy	0.94
	Chronic otitis media	0.92
	Hearing loss	0.92
	Eustachian tube dysfunction	0.88
	Otitis media with effusion	0.82
	Schwannoma	0.74
	Acute otitis media	0.50
	Normal findings	0.18

Looking at the agreement of individual classes, some classes had a higher agreement in Top-2 than Top-1. This is due to high interconnection between different conditions. In these cases, the diagnosis often relies on the physician’s experience and personal tendencies. For example, tinnitus is often present with hearing impairment; therefore, it is up to the physician to diagnose the patient with tinnitus or hearing impairment. This flexibility reduced the first-line diagnostic agreement but had a higher agreement when first-line and second-line diagnoses were considered together.

### Unattended (Automated) Versus Contact Medicine and Asynchronous Telemedicine Diagnoses

With an experimental classifier that is based on automated otoendoscopic image classification and a decision tree, Top-1 accuracy was 69.5%. The Top-1 results were compared to the initial impression of the telemedicine scenario to examine how similar the computer systems and the physicians’ predictions were. This comparison yielded a κ of 0.70, indicating substantial agreement. We measured the total execution time (loading the patient’s information file, classifying the image, and writing the prediction to a file) seven times. It took an average of 15.9 (SD 0.2) seconds to diagnose all 177 patients, which is equivalent to an average of 667.9 (SD 8.3) diagnoses per minute. All predictions and ground-truth labels are presented as a confusion matrix in Multimedia Appendix 1.

## Discussion

### Principal Results

When diagnosed by four otolaryngologists, the interrater agreement was substantial (κ=0.71) and almost perfect (κ=0.91) in Top-1 and Top-2 diagnoses, respectively. Diagnostic accuracy was stable across different survey participants, especially in Top-2. In real-world clinics, physicians often make more than one differential diagnosis at the initial visit, so it is rational to regard the Top-2 accuracy as a reliable metric. Since by its nature, asynchronous telemedicine does not permit additional interviews with the patients, information may be limited compared to traditional synchronous telemedicine or walk-in clinic visits; under such circumstances, the clinical experience might become a more critical key factor for an accurate diagnosis.

The automated diagnosis system’s Top-1 accuracy was 69.50%. Although this is acceptable when compared to other groups’ Top-1 accuracies, in real-life clinics, second or third likely differential diagnoses are essential for appropriate treatments. Therefore, we do not think the automated system is ready for clinical use. It needs further refinements.

We additionally tested the time for making a diagnosis as a measure of diagnostic efficiency. Often, it takes more than 10 minutes for a physician to make an initial impression of a new patient. In a study in a primary-care office, the median visit time was 15.7 minutes [16]. In synchronous telemedicine models, there is no advantage in terms of saving time for diagnosis; instead, it may cause temporal overhang in the connection between patients or centers. In this study, physicians were able to make an average of 1.55 diagnoses per minute, with Top-2 accuracy comparable to an attending physician. In the automated diagnosis model, computers diagnosed diseases with lightning speed. With modifications, we may use the system as a computer-aided diagnosis before asking for a second opinion by clinicians.

### Comparison With Prior Work and Limitations

**Figure 2:**
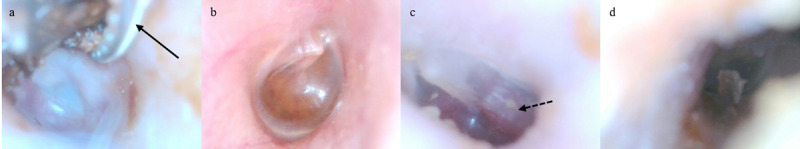
Capabilities and limitations of a consumer-grade otoendoscope images. (a) A cochlear implant electrode is exposed in the ear canal (arrow), suggesting a postoperative complication. (b) Consumer-grade otoendoscopes offer sufficient image quality to identify otitis media with effusion. (c) It is hard to diagnose middle ear tumors (dashed arrow) due to haziness of the picture. (d) The ear canal is too narrow for a consumer-grade otoendoscope to pass.

In this study, we expand the aim and scope of asynchronous telemedicine in otolaryngology by assessing all otologic diseases, rather than confining it to otoscopic findings alone as in previous studies [6,9,10]. Since we included all patients visiting our tertiary referral center, the disease spectrum was broad, including rare complications such as cochlear implant electrode exposure following surgery. Most of the middle ear diseases could be diagnosed using consumer-grade smartphone otoendoscopy and clinical manifestations, with a high degree of accuracy. Some lesions were easily identified, whereas in some cases, the consumer-grade device showed limitations associated with its resolution, contrast, or size being too large for narrow ear canals (Figure 2). With time, as technology advances, such problems might be solved. Facial palsies and external ear tumors or pits have apparent symptoms and findings, and could be diagnosed with facial photos with almost 100% accuracy. Hearing impairment and tinnitus are symptoms as well as diagnoses; if a patient claims to have it, it is likely there. However, the diagnostic accuracy of dizziness was relatively low because additional physical examinations could not be performed. In this study, since eye cameras are not currently affordable to most consumers, diagnosing dizziness was solely based on clinical representations and history, thus resulting in low diagnostic accuracy. When an eye camera or eye-tracking device becomes widely available, the feasibility of diagnosing dizziness of peripheral origin may be re-evaluated, and the overall accuracy of asynchronous telemedicine is likely to improve.

This study has some limitations. First, we could not randomly allocate the patients to telemedicine or contact medicine groups since the current legal regulations in South Korea prohibit telemedicine. However, we strictly followed the flow previously mentioned when diagnosing new patients. Second, the same attending physician with the initial impression made the final diagnosis, which may lead to bias in either the initial impression or the final diagnosis. We made sure that attending physicians did not modify the initial impression once they made an impression, and for the final diagnosis, we trusted the attending physicians to make professional clinical decisions, regardless of study enrolment or initial impressions. Last, the automated classification system was trained on images acquired by expensive otoendoscopic imaging towers (OTOLUX 0-degree telescope tethered to Olympus OTV-SP1 video imaging system), not consumer-grade images. Moreover, images of postsurgical status were not included in the original classification model; we simply used the classifying system using these lower quality otoendoscopic images. Therefore, we suspect that image quality was one of the reasons for low diagnostic accuracy in the automated diagnosis model.

### Conclusions

To our knowledge, this is the first prospective study to assess the feasibility and effectiveness of asynchronous telemedicine in otolaryngology. The findings of the study could be used in many ways, especially in the era of the COVID-19 pandemic. In offices, patients may be prediagnosed with appropriate interviews and otoscopic findings, and make all the arrangements for additional tests (hearing, balance, and imaging), if necessary, to minimize waiting time and hospital visits. In areas abundant with otolaryngologists, these may work together to draw a more accurate diagnosis by voting. Where otolaryngologists are scarce, local tele-health facilitators may interview the patients and take otoendoscopic images. These can be sent to a central server for diagnosis. Ideally, patients may also provide symptoms along with otoendoscopic images directly to a server via structured questionnaires or chatbots. Once some amount of patient’s records are stacked, otolaryngologists may remotely assess these patients in batches and provide further strategies such as observation, prescription, or recommend an office visit. Since diagnosis time is significantly reduced, it is not likely to impose a heavy burden on the existing medical resources. We claim that this approach might help alleviate the global burden of ear disease by medical resources optimization.

## Appendix

Multimedia Appendix 1 Confusion matrix (Top-1) of all classes in asynchronous telemedicine (Left) and automatic (Right). Overall accuracy: 83.05% (Left), 69.49% (Right). Contact medicine was considered a ground-truth label. AOM: acute otitis media or myringitis; BPPV: benign paroxysmal positional vertigo; Cer: cerumen impaction; COE: chronic otitis externa; COM: chronic otitis media; ETD: eustachian tube dysfunction; Ext: external ear disease (includes preauricular fistula and otohematoma); FP: facial palsy (Bell palsy); HL: hearing loss (includes conductive, sensorineural, and mixed); MD: Meniere disease; MET: middle ear tumor; Nor: normal finding; OME: otitis media with effusion; POC: postoperative complication; SCDS: superior semicircular canal dehiscence syndrome; Sch: schwannoma (vestibular and facial); S-SNHL: sudden sensorineural hearing loss; Tin: tinnitus; Tr: traumatic eardrum perforation; VN: vestibular neuritis, vestibulopathy.
