# Supplementary figures and images for: Feasibility of Asynchronous and Automated Telemedicine in Otolaryngology: Prospective Cross-Sectional Study
Source: JMIR Med Inform. 2020 Oct 19;8(10):e23680. doi: 10.2196/23680 (PMC7575342; doi:10.2196/23680)

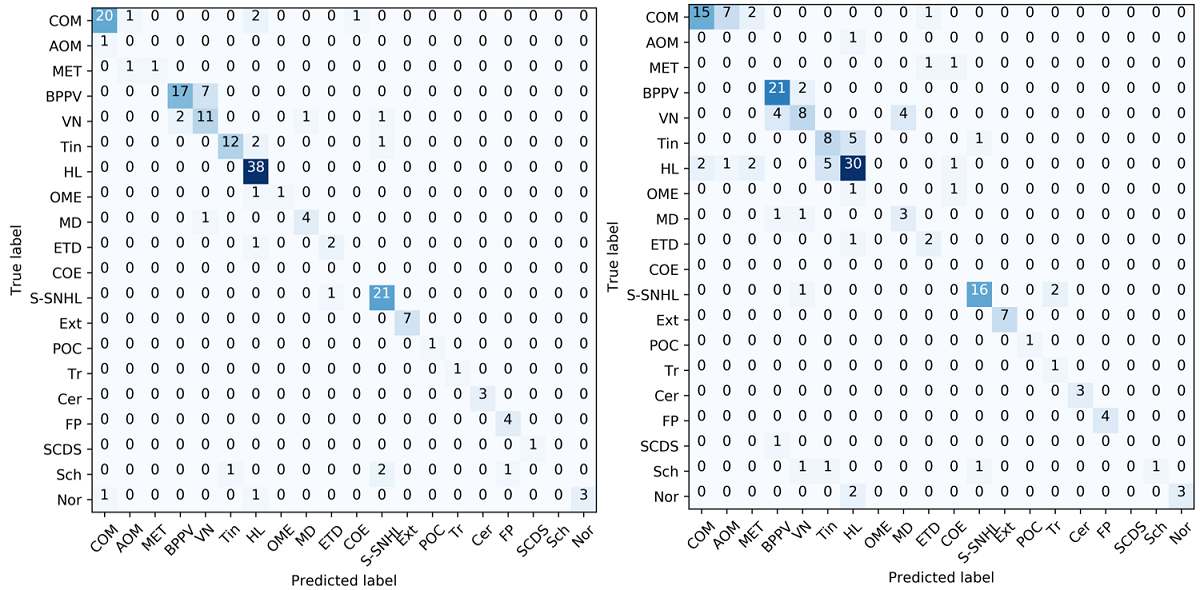

Supplement: Multimedia Appendix 1 [file medinform_v8i10e23680_app1.png]
